# Gut microbiota, microbiota‐derived metabolites, and graft‐versus‐host disease

**DOI:** 10.1002/cam4.6799

**Published:** 2024-01-18

**Authors:** XiaoYan Yue, Hongyu Zhou, ShuFen Wang, Xu Chen, HaoWen Xiao

**Affiliations:** ^1^ Department of Hematology, Sir Run Run Shaw Hospital Zhejiang University School of Medicine Hangzhou China

**Keywords:** allogeneic hematopoietic stem cell transplantation, graft‐versus‐host disease, gut microbiota, microbiota‐derived metabolites

## Abstract

Allogeneic hematopoietic stem cell transplantation is one of the most effective treatment strategies for leukemia, lymphoma, and other hematologic malignancies. However, graft‐versus‐host disease (GVHD) can significantly reduce the survival rate and quality of life of patients after transplantation, and is therefore the greatest obstacle to transplantation. The recent development of new technologies, including high‐throughput sequencing, metabolomics, and others, has facilitated great progress in understanding the complex interactions between gut microbiota, microbiota‐derived metabolites, and the host. Of these interactions, the relationship between gut microbiota, microbial‐associated metabolites, and GVHD has been most intensively researched. Studies have shown that GVHD patients often suffer from gut microbiota dysbiosis, which mainly manifests as decreased microbial diversity and changes in microbial composition and microbiota‐derived metabolites, both of which are significant predictors of poor prognosis in GVHD patients. Therefore, the purpose of this review is to summarize what is known regarding changes in gut microbiota and microbiota‐derived metabolites in GVHD, their relationship to GVHD prognosis, and corresponding clinical strategies designed to prevent microbial dysregulation and facilitate treatment of GVHD.

## 1 INTRODUCTION

Allogeneic hematopoietic stem cell transplantation (allo‐HSCT) is currently the main method for curing hematologic malignancies. However, the application of allo‐HSCT is limited by major life‐threatening complications, including graft‐versus‐host disease (GVHD), recurrence, infection, and secondary malignancies.^1^, ^2^, ^3^, ^4^ GVHD, the most common complication following allo‐HSCT, is a clinicopathological syndrome caused by donor‐derived lymphocytes attacking the organs of the transplant recipient during the process of immune reconstruction. This is mainly manifested as tissue inflammation and/or fibrosis on the skin, gastrointestinal (GI) tract, liver, lung, and on mucosal surfaces.^5^ According to statistics, the incidence of GVHD in patients receiving allo‐HSCT can reach 40%–60%, and the mortality rate can be as high as 15%.^4^, ^6^ Therefore, GVHD significantly reduces the survival rate and quality of life of transplantation patients, and has become the biggest obstacle to transplantation.^6^, ^7^, ^8^

The human body is home to trillions of microbes, most of which live within the gut. Gut microbiota and microbiota‐derived metabolites can maintain intestinal homeostasis and epithelial integrity, help resist pathogen attack, regulate immune system development and immune response, and maintain body health.^9^, ^10^, ^11^ The gut microbial balance can be disturbed by a variety of factors, leading to a significant decrease in microbial diversity and functional richness, which further contribute to various digestive system, autoimmune, metabolic, cardiovascular, neoplastic, and neuropsychiatric diseases.^12^, ^13^, ^14^, ^15^, ^16^

Recent development of new high‐throughput sequencing technologies, including metagenomics, metabolomics, and metatranscriptomics, has facilitated new research on gut microbiota, the relationship between gut microbiota or microbiota‐derived metabolites and GVHD.^17^ Meanwhile, the GI tract is the main organ targeted by GVHD. Conditioning regimens before transplantation and activation of allogeneic T cells following transplantation can damage intestinal epithelial cells (IECs) and change the composition of gut microbiota, thereby leading to gut microbiota dysbiosis and translocation.^3^, ^18^, ^19^ In addition, imbalances of gut microbiota and microbiota‐derived metabolites have been shown to play a crucial role in the occurrence and development of GVHD and are associated with lower long‐term survival and higher transplant‐related mortality (TRM).^3^, ^18^, ^19^, ^20^

Therefore, the purpose of this review is to summarize the relationship between changes in gut microbiota and microbiota‐derived metabolites in GVHD, their relationship to GVHD prognosis, and corresponding clinical strategies for microbial prevention and treatment of GVHD.

## 2 GVHD

GVHD is caused by the allogenic activation of T cells, which recognize host antigens as foreign, thereby causing an autoimmune‐like attack in various recipient organs, including the skin, lung, liver, intestine, thymus, hematopoietic system, and central nervous system (CNS).^21^ In the past, GVHD was divided into acute GVHD (aGVHD) and chronic GVHD (cGVHD) according to the time of onset. The current consensus criteria of the National Institutes of Health define different GVHD syndromes according to their clinical manifestations. Here, GVHD is subdivided into classic aGVHD, defined as an occurrence within 100 days (+100 days) after transplantation, and mainly manifests as an inflammatory reaction in the skin, GI tract, and liver. Late‐onset acute GVHD refers to GVHD featuring the clinical manifestations of classic aGVHD and occurs after +100 days. Further subcategories include new‐onset aGVHD, which occurs after +100 days, reactivation of controlled classic aGVHD after +100 days, and classic aGVHD that lasting after +100 days. Typical cGVHD, which has definite features but lacks acute features, can occur at any time after transplantation. Finally, overlapping cGVHD is diagnosed when features of both aGVHD and cGVHD coexist.^19^, ^22^, ^23^

The pathophysiology of aGVHD is divided into three main stages. The first stage involves host tissue damage caused by the preconditioning process, which in turn causes the release of inflammatory factors, chemokines, and bacterial products (e.g., lipopolysaccharides) as well as the activation of recipient antigen‐presenting cells (APCs). In the second stage, activated APCs activate donor T cells, which leads to the proliferation and differentiation of donor T cells into different subsets, including Helper T (Th) cells such as Th1, Th2, and Th17 cells, as well as CD8^+^ T cells. In the third stage, Th cells and cytotoxic T (Tc) cells work together to mediate damage to aGVHD target organs—including the intestine, liver, lungs, and skin—by secreting a variety of cytokines.^5^, ^24^ Compared to aGVHD, the pathological mechanism of cGVHD is less well understood. At present, the pathophysiology of cGVHD can be roughly divided into three stages. The first stage is characterized by the early acute inflammatory response caused by tissue damage. The second stage is characterized by chronic inflammation, which leads to damage to the thymus and to T‐ and B‐cell immune disorders. Thymus damage greatly reduces the number of thymus‐derived Treg cells, thereby resulting in loss of peripheral immune tolerance in cGVHD patients. T cells differentiate into Th1, Th2, and Th17 in response to the action of APCs, chemotaxis, infiltration, amplification, and activation to target organs, and secrete a variety of pro‐inflammatory cytokines, which leads to inflammatory damage to target organs. The third stage involves imbalances in the immune response that lead to abnormal tissue repair mechanisms, thereby causing organ damage, antibody accumulation, and tissue fibrosis. cGVHD has more complex clinical manifestations and affects numerous organs, including the skin, oral mucosa, eyes, liver, lung, and GI tract, among others.^5^, ^6^

## 3 GUT MICROBIOTA AND MICROBIOTA‐DERIVED METABOLITES

A healthy human body is inhabited by 10^13^–10^14^ different types of microbiota, including bacteria, fungi, and viruses.^25^ Most of the microbiota is colonized in the intestine, where it is known as the gut microbiota. Under physiological conditions, the gut microbiota is highly diverse and is mainly composed of *Firmicutes*, *Bacteroidetes*, *Actinomycetes*, and *Proteobacteria*. It is involved in several important physiological processes, including host nutrient absorption, material metabolism, and immune defense.^26^, ^27^ The gut microbiota plays an important role in the production of bioactive metabolites.^28^ Specifically, the microbiota consumes undigested food eaten by the host and excretes IECs as substrates. These then carry out complex and active metabolic reactions within the intestine to produce a variety of small molecule metabolites, including short‐chain fatty acids (SCFAs), bile acids (BAs), and tryptophan and its derivatives (e.g., indole and indole derivatives), among others.^29^, ^30^ These bioactive metabolites can directly or indirectly affect the physiological functions of the host, including host body development, digestion and metabolism, and immune regulation.^11^

## 4 GUT MICROBIOTA AND GVHD

The delicate balance between the human host and gut microbiota must be actively maintained by both parties to achieve a healthy steady state. Disruptions to the gut microbial balance can lead to the loss of multiple host functions, including impaired intestinal barrier function, immune dysfunction, and inflammation, which are associated with various diseases.^9^, ^11^, ^14^, ^15^ In allo‐HSCT patients, a pretreatment regimen before transplantation including chemotherapy, radiotherapy, immunotherapy, and broad‐spectrum antibiotics followed by the activation of allogeneic T cells following transplantation can damage IECs and change the composition of the gut microbiota, resulting in decreased intestinal commensal bacteria.^3^, ^31^, ^32^, ^33^, ^34^ The interaction between gut microbiota and GVHD has been studied for several decades. As early as the 1970s, it was shown that rearing mice in a sterile environment^35^ or antibiotic‐mediated intestinal purification^36^ reduced aGVHD symptoms. However, multiple subsequent clinical sample studies confirmed that reduced gut microbial diversity in allo‐HSCT patients after transplantation is associated with a significant increase in the risk of GVHD.^3^ Therefore, changes in gut microbial diversity and composition play an important role in the occurrence and development of GVHD and therefore can be used as a prognostic indicator for GVHD patients.

## 5 CHANGES IN GUT MICROBIAL DIVERSITY AND GVHD

Studies have shown that changes in gut microbial diversity are significantly associated with the occurrence, development, and prognosis of GVHD,^37^ and have confirmed that decreased gut microbial diversity after allo‐HSCT was significantly associated with shortened overall survival (OS), increased GVHD‐related mortality, and increased risk of developing GVHD.

Taur et al. found that patients with lower gut microbial diversity had significantly worse mortality outcomes, with 3‐year OS values of 36%, 60%, and 67% for patients with low, intermediate, and high gut microbial diversity groups after allo‐HSCT, respectively (*p =* 0.019).^18^ In addition, Han et al.,^38^ ILETT et al.,^39^ and Greco et al.^40^ all confirmed that aGVHD patients showed lower microbial diversity than non‐aGVHD patients, as well as that lower microbial diversity was an independent risk factor for aGVHD.^40^ A recent multicenter study also demonstrated that the gut microbial diversity of allo‐HSCT patients was lower than healthy controls and that lower gut microbial diversity was in turn associated with an increased incidence of acute intestinal GVHD (*p =* 0.02) and TRM (*p* = 0.03).^41^ Jenq et al. also demonstrated that decreased gut microbial diversity was associated with increased GVHD‐related mortality and lower OS.^42^ Another study confirmed that lower gut microbial diversity was associated with decreased OS, a higher risk of TRM (82/349 vs. 52/354, HR = 0.63, 95%CI: 0.44–0.89), and a higher risk of GVHD‐related mortality (17/244 vs. 26/184, HR = 0.49, 95%CI: 0.26–0.90).^43^

Taken together, these studies confirm that allo‐HSCT patients show significant decreases in gut microbial diversity, especially in patients with GVHD, and that such reductions in gut microbial diversity are associated with poor OS, higher incidence of GVHD, and higher GVHD‐related deaths. Accordingly, gut microbial diversity can be used as an important factor for predicting the prognosis of GVHD patients **(**Table 1 **)**.

**TABLE 1:** Summary of the relationship between gut microbiota and GVHD.

Patients	Analysis methods	Microbiota diversity	Alteration of microbiota composition	Relationship with GVHD
18 adult patients^33^	16S rRNA	↓	In GVHD mice and patients: *Lactobacillales* ↑ *Clostridiales*↓	Increased microbial chaos early after allogeneic BMT is a potential risk factor for subsequent GVHD
80 adult patients^18^	16S rRNA	/	In GVHD patients: Enterococcus, Streptococcus, Enterobacteriaceae (*Escherichia* and *Kluyvera*), and *Lactobacillus* ↑	Reduced intestinal flora diversity was associated with poorer OS, higher transplant‐related mortality, and GVHD‐related mortality
31 adult patients^52^	16S rRNA	↓	In aGVHD patients: Enterococci (*E. faecium* and *E. faecalis*) ↑ Firmicutes (Clostridia, *E. rectale*) ↓	In allo‐HSCT patients, early microbiome shifts and loss of intestinal microbiome diversity may influence intestinal inflammation
64 adult patients^42^	16S rRNA	/	In aGVHD patients: *Blautia*↓	Increased abundance of commensal bacteria belonging to the *Blautia* genus was associated with reduced GVHD‐related mortality and improved OS
66 adult patients^173^	16S rRNA	↓	In severe aGVHD patients: Bacteroides genus (*B. thetaiotaomicron*, *B. ovatus*, and *B. caccae*), Lachnospiraceae (*B. luti*) and Butyricicoccus ↓ Bacteroides genus (*B. dorei*), oral *Actinobacteria* and oral *Firmicutes*↑	Fecal microbiota at neutrophil recovery post‐HSCT is a predictor of severe aGVHD
29 pediatric patients^174^	16S rRNA and metagenomic shotgun sequencing	/	In GVHD patients: Anti‐inflammatory Clostridia (AIC) ( *Clostridiaceae* , *Ruminococcaceae*, *Blautia*, *Lachnospiraceae* )↓ *Enterococcus* , *Enterobacteriaceae*, and *Neisseriaceae* ↑	Loss of AIC due to exposure to antianaerobic antibiotics (e.g., clindamycin) is associated with the development of GVHD
81 adult patients^38^	16S rRNA	↓	In aGVHD patients: *Clostridia* (the Lachnospiraceae and Ruminococcaceae families)↓ gamma‐proteobacteria (the Enterobacteriaceae family)↑	Low microbial diversity was an independent risk factor for aGVHD, and intestinal microbiota might induce aGVHD by influencing the Treg/Th17 balance
141 adult patients^49^	16S rRNA	↓	In aGVHD patients: Clostridia (*Blautia*, Eubacterium, Lachnospiraceae, Peptostreptococcaceae, Erysipelotrichaceae, Lachnoclostridium, Erysipelatoclostridium)↓ Proteobacteria (gamma‐proteobacteria, Enterobacteriaceae)↑	The AIM score defined as microbiota diversity and gradient was positively correlated with aGVHD grade and could predict the development of aGVHD. The composition of intestinal flora at neutrophil engraftment may predict the development of aGVHD
44 adult patients^41^	16S rRNA	↓	In allo‐HSCT patients: Pseudobutyrivibrio and Subdoligranulum↓ Enterococcus↑	A lower Shannon diversity index at the time of engraftment was associated with increased incidence of acute intestinal GVHD and transplant‐related deaths
1325 adult patients^51^	16S rRNA	/	In GVHD mice and patients: Enterococcus (*E. faecium*)↑	Fecal domination by Enterococcus was associated with significantly reduced OS, increased GVHD‐related mortality, and increased risk of moderate‐to‐severe acute GVHD
70 adult patients^73^	16S rRNA	↓	In aGVHD patients: Lachnospiraceae, Ruminococcaceae, *Blautia*↓	Microbiota alterations were associated with the severity of gastrointestinal aGVHD
1362 adult patients^43^	16S rRNA	↓	In aGVHD patients: *Enterococcus*, *Klebsiella*, *Escherichia*, *Staphylococcus*, and *Streptococcus*↑	Low microbial diversity was associated with significantly reduced OS, higher risks of transplant‐related mortality and GVHD‐related mortality
163 adult patients^39^	Shotgun metagenomic sequencing	↓	In allo‐HSCT patients: *Staphylococcus*, *Eggerthella*, *Streptococcus*, and *Lactobacillus*↑ In aGVHD patients: *Blautia* and *A. muciniphila*↓	The absence of *Blautia* and *Bacteroides* was associated with an increased odds of aGVHD, whereas *E. faecium* was associated with a decreased odds of aGVHD
1081 adult patients (54 patients with cGVHD)^48^	16S rRNA	↓	In cGVHD patients: high abundances of the genus *Akkermansia* were associated with cGVHD development, while low abundance of *Lachnoclostridium* was associated with remaining cGVHD‐free	The predictive model demonstrated that the lower abundances of butyrate‐producing genera *Lachnoclostridium*, *Clostridium*, and *Faecalibacterium* were associated with reduced incidence of cGVHD, while the higher abundances of *Akkermansia* and *Streptococcus* were associated with decrease incidence of cGVHD
100 adult patients^40^	16S rRNA	↓	In aGVHD patients: Lachnospiraceae↓ Enterococcaceae and Staphylococcaceae↑	Low microbial diversity was significantly associated with increased risk of grade II‐IV and III‐IV aGVHD. The presence of Lachnospiraceae was associated with a reduced risk of developing aGVHD, while the relative dominance of Enterococcaceae and Staphylococcaceae was associated with an increased incidence
42 pediatric patients^175^	16S rRNA	↓	In aGVHD patients: Pasteurellales and Pasteurellaceae↑	Gut microbiota diversity was lowest in gut aGVHD, which was consistent with higher mortality rate, greater treatment difficulty, and worse OS
55 adult patients^176^	16S rRNA	↓	In grade 3 aGVHD patients: *Veillonella*↑ In extensive cGVHD patients: *Faecalibacterium*, *Coprococcus*, and *Bifidobacterium*↓	These long‐term gut microbial alterations may be associated with the development and exacerbation of late complications in post‐transplant survivors
226 adult patients^47^	16S rRNA	↓	In aGVHD patients: *Clostridia* (*Blautia*, *Eubacterium*, *Coprococcus*, and *Ruminococcus*), butyrate producers, and lower ratios of strict‐to‐facultative anaerobic bacteria (S/F anaerobe ratio)↓	Higher abundance of *Clostridia*, butyrate producers, and S/F anaerobe ratio were predictors of longer overall survival and decreased risk of GVHD‐related death

## 6 ASSOCIATION BETWEEN CHANGES IN THE COMPOSITION OF THE GUT MICROBIOTA AND GVHD

Dysbiosis of the gut microbiota is manifested by altered composition of the microbiota, and is significantly associated with GVHD.^37^ Studies have indicated that GVHD is associated with decreased abundance of specific bacteria, including *Firmicutes (Clostridium, Faecalibacterium, Lachnospiracea, Ruminococcaceae, Eubacteriaceae* and *Peptostreptococcaceae), Bacteroidetes* (*Bacteroides* and *Parabacteroides)*, and *Actinobacteria*, as well as an increase in the abundance of *Proteobacteria* (*Gamma‐proteobacteria* and *Enterobacteriales*), *Verrucomicrobia* (*Akkermansia*) and opportunistic pathogens belonging to the *Firmicutes* (*Lactobacillus, Staphylococcaceae, and Enterococcus)*.^39^, ^40^, ^42^, ^44^, ^45^, ^46^

Related studies have shown that the abundance of *Clostridia*, *Lachnospiraceae, Blautia*, *Bacteroide*, and *Akkermansia muciniphil* significantly decreased in aGVHD patients, which was associated with lower OS, and higher GVHD‐related mortality.^39^, ^47^ In cGVHD patients, it was also found that reduced abundance of butyrate‐producing bacteria such as *Lachnoclostridium*, *Clostridium*, and *Faecalibacterium* was associated with a higher incidence of cGVHD.^48^ In another study, reduced accumulated intestinal microbiota (AIM) scores 15 days after transplantation, which reflect the abundance of four focal bacterial clades (i.e., *Lachnospiraceae, Peptostreptococcaceae, Erysipelotrichaceae*, and *Enterobacteriaceae*), were an independent risk factor for aGVHD. Moreover, AIM scores were positively correlated with aGVHD grade and could therefore be a valuable predictor for the risk of the development of aGVHD.^49^ In contrast, the abundance of *Lactobacillales, Staphylococcaceae, Enterobacteriales*, and *Enterococcus* has been found to be significantly increased in aGVHD patients, which was associated with the severity and risk of aGVHD and cGVHD, lower OS, and increased GVHD‐related mortality.^43^, ^48^, ^50^, ^51^, ^52^ Moreover, gut microbial biomarkers have been found to be able to predict the risk of organ‐specific aGVHD involvement, with patients showing relative intestinal dominance of *Staphylococcaceae* (>40%) had a higher risk of aGVHD with liver involvement (early: HR = 7.99, *p* = 0.007; late: HR = 5.14, *p* = 0.029), early GI involvement (HR = 4.84, *p* = 0.037), and the occurrence of steroid‐refractory aGVHD (RR = 8.40, *p* = 0.007).^40^

Changes in the composition of gut microbial may contribute to the imbalance of specific immune cell subsets, which are involved in GVHD pathogenesis. For example, Han et al. demonstrated that decreased abundance of *Lachnospiraceae* and *Ruminococcaceae* and increased abundance of *Enterobacteriaceae* were associated with Treg/Th17 imbalance, which might be through acetylated H3 in CD4^+^ T cells. Therefore, gut microbial might induce aGVHD by influencing the Treg/Th17 balance.^38^ In addition, using a cGVHD mouse model, bacterial extracts of gut microbiota from cGVHD patients were found to induce the murine splenic T cells to differentiate into Th1 cells and inhibit their differentiation to Treg cells, resulting in Th1/Treg imbalance, which was significantly correlated with the onset of cGVHD.^53^ Therefore, these studies have shown that gut microbial imbalance can lead to immune imbalance, which in turn participates in the pathogenesis of GVHD.

In conclusion, the studies listed above suggest that changes in gut microbial composition were significantly correlated with the prognosis of GVHD patients (Table 1) and could be used as biomarkers and therapeutic targets for predicting GVHD.

## 7 GUT MICROBIOTA‐DERIVED METABOLITES AND GVHD

**FIGURE 1:**
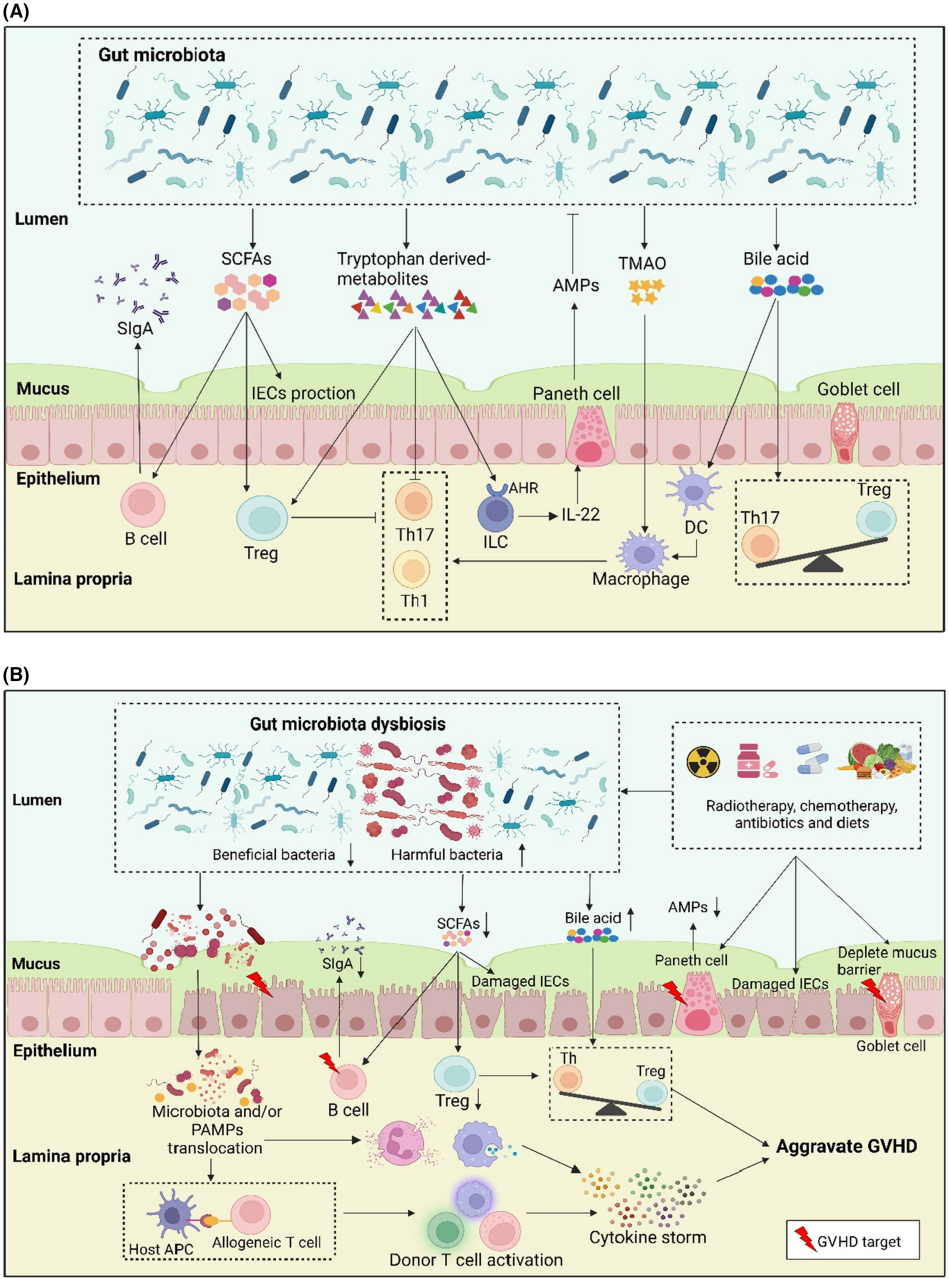
Gut microbiota, microbiota‐derived metabolites, and graft‐versus‐host disease. (A) Gut microbiota, microbiota‐derived metabolites in homeostasis. In a stable state, a large number of gut microbiota are distributed in the intestinal lumen, which can maintain intestinal homeostasis and epithelial integrity, resist pathogens, regulate immune system development and immune response. Microbiota‐derived metabolites such as SCFAs, tryptophan and its derivatives, and bile acids can directly act on intestinal epithelial cells and intestinal immune cells to regulate the differentiation, recruitment and activation of immune cells. The intestinal epithelial surface maintains a complete barrier to prevent bacteria from invading host tissues, Paneth cells produce AMPs to prevent microbial invasion, goblet cells produce a mucus barrier that separates bacteria from epithelial cells and the lymphocytes (including T cells, B cells, ILC, and MAIT cell) distributed in the intestinal lamina propria resist the invasion of pathogenic microbiota and inhibit the immune response. (B) Relationship between gut microbiota and pathogenesis of GVHD. Preconditioning regimens (including radiotherapy, chemotherapy, immunotherapy, and broad‐spectrum antibiotics) before allo‐HSCT can damage IECs, Paneth cells, and goblet cells, thereby damaging the intestinal barrier, reducing the production of AMPs, depleting the mucus barrier, leading to the dysbiosis of gut microbiota and the translocation of gut microbiota. Gut microbiota, microbiota‐derived metabolites (PAMPs, such as bacterial lipopolysaccharide) are translocated to the lamina propria of the intestine and are recognized by host APCs. APCs can activate allogeneic reactive T cells, lead to the continuous proliferation of autologous reactive T cells, release a large number of cytokines, trigger cytokine storms, and aggravate intestinal tissue damage. B cells are considered to be the target of GVHD, and their damage can lead to a decrease in sIgA secretion and aggravate intestinal inflammation and bacterial translocation. The imbalance of gut microbiota leads to the disorder of its metabolites, which is mainly manifested by the decrease of SCFAs, tryptophan and its derivatives, and the increase of bile acids, which can aggravate the damage of intestinal epithelial cells, lead to the imbalance of immune cells in intestinal lamina propria, and aggravate GVHD. AhR, aryl hydrocarbon receptor; allo‐HSCT, allogeneic hematopoietic stem cell transplantation; AMPs, antimicrobial peptides; APCs, antigen‐presenting cells; DC, dendritic cell; GVHD, graft‐versus‐host disease; IECs, intestinal epithelial cells; IgA, immunoglobulin A; ILC, innate lymphoid cell; MAIT, mucosal‐associated invariant T cell; PAMPs, pathogen‐associated molecular patterns; SCFAs, short chain fatty acids; Th1, T helper 1 cell; Th17, T helper 17 cell; TMAO, trimethylamine N‐oxide; Treg, regulatory T cell.

Gut microbiota exert effects on tissues mainly through microbiota‐derived metabolites. Accordingly, changes in the gut microbiota can lead in turn to changes in microbiota‐derived metabolites. Studies have shown that these metabolites play a crucial role in regulating intestinal homeostasis and the immune response. Moreover, they mediate how IECs and intestinal immune cells maintain intestinal barrier function and host immune response (Figure 1A).^54^, ^55^, ^56^ Microbiota‐derived metabolites which include SCFAs, tryptophan and its derivatives, choline metabolites, tyrosine, and BAs can affect the severity and prognosis of GVHD (Table 2, Figure 1B).^57^, ^58^

**TABLE 2:** Gut microbiome metabolites and GVHD.

Gut microbiome metabolites	Change	Mechanism of action	Impact on GVHD
SCFAs: butyrate and propionate^48^, ^57^, ^73^, ^75^	Decrease	Regulate intestinal immunity, enhance epithelial cell barrier function	Exogenous butyrate supplementation can reduce the severity of aGVHD and improve prognosis
3‐indoxyl sulfate (3‐IS)^96^	Decrease	/	Lower urinary 3‐IS were associated with significantly higher transplant‐related mortality and lower overall survival due to increased intestinal GVHD
Indole‐3‐carboxaldehyde (ICA)^58^	/	Limit intestinal epithelial injury, reduce bacterial migration, reduce inflammatory cytokine production	ICA treatment can alleviate aGVHD pathology and reduce aGVHD mortality
Indoleamine 2,3‐dioxygenase (IDO)^76^	Increased in IECs of GVHD mice	Reduce the proliferation and survival of T cells	Regulation of IDO pathway can reduce colon inflammation, GVHD severity, and GVHD‐related mortality
Trimethylamine N‐Oxide (TMAO)^102^	Elevated in mice with high choline diet	Enhanced M1 polarization via activating the NLRP3 inflammasome and activate Th1 and Th17 differentiation and proliferation	Administration of TMAO aggravated the severity and mortality of GVHD mice
Tyrosine^103^	Decrease	Regulating intestinal microbiome and metabolome	Exogenous tyrosine supplementation can improve overall survival and ameliorate symptoms at the early stage of aGVHD
Bile acids: taurine^116^	Increase	Stimulate NLRP6 in IECs	Augment GVHD severity in mice

## 8 SCFAs

SCFAs are produced by gut microbiota fermenting food that the host cannot metabolize, and include butyrate, propionate, and acetate, among others.^59^ SCFAs are an important energy source for gut microbes, as well as for the IECs. In addition to serving as a local substrate for energy production, SCFAs also have immunomodulatory functions, and play important roles in innate immunity, and the production of lymphocytes, neutrophils, monocytes, macrophages, and dendritic cells (DCs). Moreover, they also possess anti‐inflammatory effects as histone deacetylase (HDAC) inhibitors.^54^, ^60^, ^61^, ^62^, ^63^ Studies have also shown that SCFAs can promote the expression of genes such as aryl hydrocarbon receptor (AhR) and hypoxia‐inducible factor 1α (HIF1α); this occurs via the activation of cell surface G‐protein receptor 41 (GPR41) receptor and the inhibition of HDAC, which promotes the production of IL‐22 by CD4^+^ T cells and innate lymphoid cells (ILCs).^64^ SCFAs are also known to induce the production and enhance the function of Treg cells by inhibiting HDAC, which occurs by promoting the acetylation of histone H3. This in turn triggers the upregulation of FOXP3 and induces the proliferation of Treg cells by activating the GPR43 receptor, which promotes IL‐10 release.^65^, ^66^, ^67^, ^68^ Furthermore, SCFAs have been found to inhibit the maturation of DCs and affect the secretion of cytokine IL‐23,^69^ which regulates the function of macrophages and the secretion of cytokines.^70^ Finally, SCFAs have multiple effects on IECs and intestinal immune cells, insofar as they maintain IEC barrier integrity and prevent the translocation of pathogenic bacteria.^71^, ^72^

Many studies have confirmed that SCFA levels were significantly reduced in GVHD patients and were associated with GVHD severity and mortality. A study of 316 patients undergoing allogeneic HSCT found a significant reduction in SCFA production at the onset of aGVHD, with variation depending on disease severity. In patients with severe aGVHD, the levels of acetate, propionate, and butyrate decreased by 75.8%, 95.8%, and 94.6%, respectively. Moreover, patients with mild aGVHD showed a decrease in butyrate of 86.0% but patients with severe aGVHD showed decreases as great as 94.6%. These data suggest that butyrate can be used as a potential diagnostic marker for aGVHD patients.^73^ In a clinical trial, plasma butyrate concentration was also tracked along with urinary‐derived human chorionic gonadotropin (uhCG) during the treatment of steroid‐refractory aGVHD. Compared to the baseline condition, uhCG responders showed higher plasma butyrate concentrations on Days 28 and 56, while no change in butyrate concentration over time was observed in non‐responders.^74^ SCFAs were also found to be less abundant in cGVHD patients. In addition, Markey et al. found that compared to patients without cGVHD, the plasma concentration of SCFAs (including butyrate and propionate) in cGVHD patients significantly decreased within 100 days of transplantation.^48^

At present, the specific mechanism by which SCFAs contribute to GVHD remains unknown. However, Mathewson et al. found that butyrate was significantly reduced in aGVHD mice model, and the reduction of butyrate in CD326^+^ IECs following allo‐HSCT can lead to a decrease in histone acetylation. Interestingly, IEC acetylation levels can be restored by supplementation with exogenous butyrate, which directly enhances the barrier function of epithelial cells and reduces aGVHD via a Treg‐independent pathway. In addition, alteration of the indigenous microbiota in the host to produce high levels of butyrate can also been found to reduce the severity of aGVHD.^75^ Herefore, this study suggests that local and specific alterations in microbial metabolites may confer direct beneficial effects on GVHD target tissues and can mitigate the severity of aGVHD. In another study, indoleamine‐2,3‐dioxygenase (IDO) expression was found to be upregulated in intestinal parenchymal cells and APCs in mouse via IFN‐γ produced by alloreactive T cells. Butyrate acts as a histone deacetylase (HDAC) inhibitor and effectively reduces GVHD by inhibiting indoleamine‐2,3‐dioxygenase (IDO)‐dependent innate immune and allo‐stimulating APC functions in a STAT‐3‐dependent manner.^4^, ^76^, ^77^ Butyrate is also known to promote histone H3 acetylation by inhibiting HDACs, up‐regulating FOXP3 expression, promoting the differentiation of Tregs, and enhancing Treg function.^68^ Tregs have been found to be highly efficient during the prevention and treatment of GVHD.^78^ SCFAs can affect the host through GPR43, which is expressed in a variety of cells, including IECs. Another study using a mouse model demonstrated that butyrate and propionate both reduce intestinal permeability and relieve GVHD by binding to the GPR43 receptor on IECs and thereby activating the NLRP3 inflammasome via ERK phosphorylation.^57^ However, signal transduction inhibitors currently approved by the Food and Drug Administration (FDA) for GVHD treatment, such as ibrutinib can inhibit BTK in B cells, leading to reduced production of autoreactive antibodies or inhibit the homologous enzyme, interleukin‐2‐inducible T‐cell kinase (ITK), leading to selective suppression of Th2 immune responses that may contribute to the pathogenesis of cGVHD,^79^, ^80^ while ruxolitinib can inhibit the production of inflammatory cytokines and regulate immune response by inhibiting JAK–STAT signaling pathway, and finally achieve the purpose of treating GVHD.^81^ Ruxolitinib has shown excellent efficacy with overall response rate (ORR) of 62.3 at Day 28 for steroid‐refractory aGVHD in REACH2 clinical trial and with ORR of 49.7% at Week 24 regardless of the cGVHD‐involved organs in patients with steroid‐refractory/intolerant cGVHD in REACH3 clinical trial.^81^, ^82^ Belumosudil, a selective inhibitor of Rho‐associated coiled‐coil‐containing protein kinase 2 (ROCK2) has emerged as a promising novel therapeutic approach for treating steroid‐refractory/intolerant cGVHD with 74%–76% ORR.^83^ SCFAs can improve GVHD by inhibiting HDAC activity and binding to GPR43, which differs from other known mechanisms of these signal transduction inhibitors, and suggests that SCFAs may provide a new treatment option for GVHD patients.

However, Golob et al. noted that although butyrate may help prevent the occurrence of aGVHD, once aGVHD has occurred and entered into progress, high level of butyrate may impair IEC recovery and actually exacerbate aGVHD.^84^ The study also found that the presence of butyrate‐producing bacteria during 21 days after onset of severe aGVHD was associated with increased risk of steroid‐resistant GVHD and cGVHD.^84^ This is primarily because butyrate inhibits the proliferation of colon stem/progenitor cells, thereby preventing colon stem cells from forming a complete monolith of the epithelium.^84^, ^85^ Therefore, if butyrate is persistent and active during the progress of severe intestinal aGVHD, loss of epithelial structure can lead to the exposure of colonic stem cells to microbially produced butyrate, which in turn impairs the recovery of the colonic mucosa of aGVHD patients, and thus leads to increased risk of refractory aGVHD and cGVHD.^84^ Therefore, supplementation with SCFAs at the time of the beginning of aGVHD may be more useful.

## 9 TRYPTOPHAN AND ITS DERIVATIVES

Tryptophan is an aromatic essential amino acid. Studies have shown that gut microbiota metabolize 4%–6% of available tryptophan into indole and indole derivatives, including indole‐3‐aldehyde (IAld), indole‐3‐acid‐acetic (IAA), indole‐3‐propionic acid (IPA), indole‐3‐acetalic acid (IAAld), and indoleacetic acid. These indoles and indole derivatives are AhR ligands, and AhRs are widely expressed in immune cells,^86^, ^87^, ^88^ since AhR signaling is an important component of the immune response at the barrier. There it helps to maintain intestinal homeostasis by promoting epithelial cell recovery, maintaining barrier integrity, and by facilitating the function of some immune cells.^89^, ^90^, ^91^

Indoles are produced by tryptophan from commensal bacteria expressing tryptophanase. They are not only an important intercellular signal for the microbial community but also play a role in regulating intestinal immune balance, inhibiting the inflammatory response, and maintaining intestinal barrier function.^92^ Studies have shown that indoles and indole derivatives can also regulate the differentiation of Treg and Th17 cells, inhibit Th17 cells, and promote Treg cell differentiation.^93^ In addition, indole derivatives produced by *Lactobacillus reuteri* have been found to promote the transformation of intestinal epithelial CD4 + T cells into CD4 + CD8 + double positive intraepithelial lymphocytes, which facilitates the maintenance of intestinal immune homeostasis.^94^ Indoles can be further metabolized into 3‐indoxyl sulfate (3‐IS) by cytochrome P450 enzymes (including CYP2E1) and sulfotransferase (SULT), which is excreted in the urine.^95^ Holler et al. demonstrated that urinary 3‐IS levels decreased in allo‐HSCT patients, but were higher in patients receiving antibiotics with GI GVHD. In addition, urinary 3‐IS levels were positive correlated with gut microbiota disruption in patients undergoing allo‐HSCT; these disruptions saw decreased abundance of some Enterococci spp. but increased abundance of colonic commensals such as *E. rectale* and *Clostridium phytofermentalis*.^52^ In another study, Weber et al. found that decreased urinary excretion of 3‐IS within the first 10 days after allo‐HSCT was associated with significantly increased TRM (*p =* 0.017) and worse OS (*p =* 0.05) within the first year following allo‐HSCT. Moreover, the composition of the gut microbiota was also found to be able to predict the level of urinary 3‐IS. The abundance of *Lachnospiraceae* and *Ruminococcaceae* (class: *Clostridia*) has also been found to be associated with higher levels of urinary 3‐IS, while the abundance of the class of *Bacilli* was correlated with lower levels of urinary 3‐IS.^96^ Taken together, these findings suggest that urinary 3‐IS levels may be an important marker of gut microbiota disruption and can reveal increased risk of developing GI GVHD following allo‐HSCT.

Indole‐3‐carboxaldehyde (ICA) is specific, biologically relevant indole derivative. In one study, Swimm et al. used a mouse model to reveal that ICA treatment can limit intestinal epithelial injury, reduce epithelial bacterial translocation, reduce inflammatory cytokine production, reduce aGVHD pathological scores, and reduce aGVHD mortality. Moreover, this is accomplished without affecting the donor T‐cell‐mediated graft‐versus‐leukemia response. Transcriptional profiling and gene ontology analysis suggest that ICA treatment can up‐regulate genes associated with type I interferon (IFN1) response, which has been reported to resist radiation‐induced intestinal injury and reduce aGVHD pathological scores. This is thought to result from the fact that the protective effect of ICA against radiation exposure is eliminated in mice lacking IFN1 signaling. Therefore, this study confirmed that indole metabolites produced by intestinal flora can limit intestinal inflammation and injuries associated with myeloablative chemoradiotherapy and aGVHD through type I IFNs. Moreover, this metabolite‐mediated effect does not affect the anti‐tumor response, or require new treatment options for BMT patients at risk of GVHD.^58^

In immune cells and epithelial cells, tryptophan enters the kynurenine pathway via indoleamine 2,3‐dioxygenase (IDO) and generates kynurenine (Kyn) and its downstream products.^87^ IDO is the rate‐limiting enzyme in the degradation of tryptophan to Kyn. It is mainly expressed by APCs and parenchymal cells and is further induced by inflammation.^97^ Studies have shown that the gut microbiota plays an important role in the activation of IDO. In addition, Jasperson et al. confirmed that IDO is a key regulator of GVHD, and that IDO expression was strongly upregulated in the colonic epithelial cells of GVHD mice. Furthermore, IDO can reduce the proliferation and survival of T cells, thereby reducing colon inflammation, GVHD severity, and GVHD‐related mortality. Therefore, this study indicated that regulating the IDO pathway is an effective method for the treatment of GVHD.^76^ Another recent study found that aGVHD is associated with significant changes in microbial‐derived metabolites, especially AhR ligands. Of these, tryptophan metabolites, including 3‐indoxyl sulfate, indole acetate, indole acetylglutamine, and indole propionate, as well as host‐derived compounds produced by the IDO tolerogenic pathway, were all significantly reduced by the onset of aGVHD.^98^ Therefore, this study suggests that the allogeneic immune response during aGVHD may be affected by the reduction of AhR ligands produced by the gut microbiota, insofar as this limits the generation of IDO and affecting the allogeneic T‐cell response.^76^, ^89^, ^98^

## 10 TRIMETHYLAMINE N‐OXIDE (TMAO)

Choline, phosphatidylcholine, carnitine, and glycerophosphocholine are highly abundant in eggs, liver, dairy products, peanuts, and other foods. In the gut, each of these compounds can be metabolized to trimethylamine (TMA), which is then converted to trimethylamine N‐oxide (TMAO) by host hepatoflavin monooxygenase. The intestinal bacteria regulating TMA generation mainly include *Campylobacter jejuni*, *Clostridium*, *Bifidobacterium*, and *Faecalibacterium prausnitzii*.^99^ As a circulating intestinal microbial metabolite, TMAOs are capable of inducing vascular inflammation and endothelial dysfunction by forming and activating the NLRP3 inflammasome in endothelial cells.^100^

Wu et al. found that TMAO or a high choline diet could aggravate disease severity and mortality in GVHD mice, and a choline structural analog known as 3,3‐dimethyl‐1‐butanol (DMB)^101^ was found to reduce elevated serum TMAO concentrations caused by a high choline diet and reverse TMAO‐induced GVHD severity and mortality. The mechanism occurs because TMAO can promote the nuclear translocation of NF‐kB and increase the production of reactive oxygen species in mitochondria, activate the NLRP3 inflammasome, promote the activation of the downstream Caspase‐1, and produce IL‐1β inflammatory factors. Taken together, these effects promote the differentiation of macrophages into the M1 phenotype in GVHD mice, where they then secrete pro‐inflammatory mediators to induce the differentiation of allogeneic T cells into Th1 and Th17 subsets; this differentiation ultimately causes worsening of GVHD.^102^

## 11 TYROSINE

Tyrosine is a non‐essential amino acid that is an important precursor for the synthesis of many bioactive substances. Huang et al. found that the abundance of *Lachnospiraceae* subclass in aGVHD mice was significantly reduced in the intestine. This was accompanied by a decrease in tyrosine content in the intestine, and multi‐component correlation analysis suggested that the presence of tyrosine was closely related to the abundance of key bacterial species in aGVHD. Moreover, by providing a safe dietary tyrosine supplement, the survival time of aGVHD mice was significantly prolonged, and intestinal rejection symptoms improved. Meanwhile, the abundance of the dominant bacterial community in the intestinal tract was found to increase following the recovery of various metabolites related to the tyrosine metabolic pathway. Thus, since mice deprived of a tyrosine diet developed more severe rejection symptoms, diet therapy may be a potential treatment for aGVHD.^103^

Gut microbiota can metabolize tyrosine into derivatives such as p‐cresol sulfate and 3‐phenylpropionate.^104^ Reikvam et al. estimated the metabolic characteristics of patients with and without aGVHD by random forest analysis of serum metabolic profiles. They found that patients with advanced aGVHD had increased levels of potential pro‐inflammatory tyrosine metabolites (i.e., p‐cresol sulfate and 3‐phenylpropionate) formed by gut microbiota, and these could be used as candidate biomarkers for predicting the risk of GVHD.^104^ Reikvam et al. performed a similar analysis of cGVHD patients in another study and found that compared with patients without cGVHD, the levels of tyrosine metabolites (including phenylacetate, 3‐(4‐hydroxyphenyl) lactate, and tyramine o‐sulfate) in the gut microbiota in serum of patients with cGVHD were higher. Thus, changes in tyrosine metabolite levels can partially reflect changes in gut microbiota composition following allo‐HSCT.^105^ However, the specific mechanism by which tyrosine and its metabolites regulate GVHD remains unclear. Accordingly, further investigation is required in future studies.

## 12 BAs AND THEIR METABOLITES

BAs are metabolites affected by intestinal microbial composition,^106^ and can be divided into primary and secondary BAs according to their source. Both primary and secondary BAs regulate the host metabolism and host immune response together.^107^, ^108^ Studies have shown that primary and secondary BAs act as signaling molecules to target BA receptors in host immune cells (e.g., farnesoid X receptor and the G‐protein‐coupled receptor TGR5),^109^ downregulate TNF‐α and IL‐12 in DCs and monocytes, and increase the production of IL‐10 in macrophages. Via these functions, they disrupt the pro‐inflammatory functions of immune cells.^110^, ^111^ In addition, BAs are involved in regulating the differentiation and function of T cells, including both Th17 and Treg cells.^112^ Hang et al. screened a library of BA metabolites and identified that derivatives of secondary BAs including lithocholic acid (LCA), 3‐oxoLCA, and isoalloLCA can regulate T‐cell function in mice. Of these BA derivatives, 3‐oxoLCA was found to inhibit Th17 differentiation by directly binding to the key transcription factor retinoid‐related orphan receptor‐γt (ROR‐γt). In contrast, isoalloLCA increased the differentiation of Treg cells by inducing oxidative phosphorylation and enhancing CNS3 H3K27 acetylation, both of which promote the expression of FOXP3. Therefore, this study indicated that BA metabolites can regulate host immunity by directly regulating the balance of Th17 and Treg cells.^113^ Furthermore, Huh et al. used both in vitro and in vivo datasets to demonstrate that the BA metabolite isoLCA inhibited the differentiation of immature CD4 + T cells into TH17 cells by inhibiting ROR‐γt.^114^

Next, Reikvam et al. analyzed serum metabolite levels in patients with and without cGVHD. Their Random Forest Classification Analysis revealed that four BAs, including the secondary BA hyocholate, as well as three primary BAs, glycochenodeoxycholate sulfate, taurocholate, and glycocholate, ranked among the 30 highest‐ranking metabolites that differed among cGVHD patients and control patients. These results show that differences in BA metabolism were found in cGVHD patients.^115^ Taurine is another BA metabolite of interest; for instance, Toubai et al. used a mouse model to reveal that taurine could activate the signal transduction of the NLRP6 inflammasome and thereby aggravate aGVHD.^116^ In addition, Michonneau et al. used a metabolomic analysis to reveal that taurine production was significantly reduced in the plasma of patients without GVHD after HSCT. In contrast, the levels of primary and secondary BAs in the plasma of aGVHD patients were significantly increased. Therefore, this study suggests that the allogeneic immune response during aGVHD may be affected by BAs, thereby limiting the production of indoleamine 2,3‐dioxygenase and affecting allogeneic T‐cell reactivity.^98^

In conclusion, these studies have confirmed that gut microbiota‐derived metabolites such as SCFAs, tryptophan, TMAO, tyrosine, and BAs play important roles in the occurrence and development of GVHD. Moreover, they represent an important opportunity for a novel therapeutic strategy for the treatment and prevention of GVHD. However, the specific mechanisms by which gut microbiota‐derived metabolites regulate GVHD is not completely clear, and further research is therefore still required.

## 13 GUT MICROBIOTA‐RELATED THERAPEUTIC OPTIONS FOR GVHD PATIENTS

Previous studies have demonstrated the importance of gut microbiota and microbiota‐derived metabolites for patients with GVHD. Therefore, purposefully regulating the gut microbiota via interventions may improve the prognosis of allo‐HSCT. Specifically, using treatments to maintain gut microbial homeostasis and preserving the advantages of beneficial bacteria may improve clinical outcomes for allo‐HSCT recipients. The main strategies used to accomplish this include nutritional support therapy, fecal microbiota transplantation (FMT), probiotics, prebiotics, and antibiotics, all of which aim to supplement or regulate the biology of the intestinal microbiome **(**Table 3 **)**.

**TABLE 3:** Summarizing the advantages and disadvantages of gut microbiota‐related therapeutic options.

	Advantages	Disadvantages
Nutritional support therapy^119^, ^125^, ^126^	EN can maintain the integrity of intestinal mucosa, regulate the composition of gut microbiota, reduce the incidence and severity of GVHD, and reduce the mortality associated with GVHD	Patients receiving PN are prone to intestinal microbiota dysregulation, increased intestinal microbiota translocation, and increased incidence, severity, and GVHD‐related mortality
Fecal microbiota transplantation^128^, ^136^, ^138^, ^140^, ^142^	FMT can directly change the composition of the gut microbiota of the host to rebuild the gut microbiota balance, repair the intestinal mucosal barrier, control the inflammatory response, and regulate the body's immunity	Drug‐resistant bacterial colonization, intestinal infection, bloodstream infections, sepsis, and even death
Probiotics^33^, ^144^, ^147^	Maintaining the balance of gut microbiome, inhibiting intestinal inflammation, protecting intestinal mucosal barrier, regulating the body's immunity, reducing the severity of GVHD, and improving survival	For patients with compromised immune function, probiotic use was associated with a higher incidence of infection
Prebiotics^152^, ^153^, ^154^	Maintain intestinal integrity, improve gut microbiome composition, promote beneficial growth, regulate immune response, reduce the severity of GVHD, and improve survival	Gastrointestinal symptoms, allergic reactions
Antibiotic^163^, ^165^, ^166^, ^167^, ^168^, ^169^	Regulate gut microbiome composition, eliminate target harmful bacteria, inhibit secondary bacterial proliferation, and reduce bacterial translocation	Some broad‐spectrum antibiotics can lead to gut microbiota imbalance, colonization of resistant bacteria, and increased incidence, severity, and GVHD‐related mortality

## 14 NUTRITIONAL SUPPORT THERAPY

Most allo‐HSCT patients develop some degree of nausea, mucositis, and anorexia due to the preconditioning regimen. Therefore, oral nutrient reduction is very common for allo‐HSCT patients.^117^, ^118^ In addition, the changes in gut microbiota observed during HSCT and GVHD may reflect the fact that insufficient nutrition to maintain a balanced gut microbiota is present in the intestine.^21^

Total parenteral nutrition (TPN) is widely used to support allo‐HSCT recipients. However, an increasing body of evidence supports the use of enteral nutrition (EN) instead. Several studies have confirmed that EN can reduce the incidence and severity of GVHD and decrease GVHD‐related mortality in allo‐HSCT patients. For example, Seguy et al. demonstrated that patients receiving EN after allo‐HSCT showed lower aGVHD incidence and mortality, while parenteral nutrition (PN) was associated with higher aGVHD incidence and mortality.^119^ Svahn et al. also demonstrated that EN deficiency was associated with a higher incidence of aGVHD and poorer OS.^120^ In addition, Gonzales et al. demonstrated that the incidence of aGVHD and non‐relapse mortality were lower in an EN group in allo‐HSCT patients undergoing myeloablative preconditioning.^121^ Another retrospective study also demonstrated that compared to adequate EN, inadequate nutrition and adequate PN both showed increased non‐relapse mortality, lower 5‐year OS, lower GVHD‐free/relapse‐free survival, and increased incidence of aGVHD.^122^ Moreover, Zama et al. conducted a meta‐analysis that confirmed that EN reduced the incidence of aGVHD, especially grade III‐IV and intestinal aGVHD.^123^ Finally, another recent study showed that patients receiving PN had a higher incidence of aGVHD than those receiving EN (9.1% vs. 24.8%, *p =* 0.01).^124^

Different nutritional methods can also affect the composition of gut microbiota. A retrospective study confirmed that the loss of *Blautia* was associated with receiving TPN for more than 10 days, and the loss of *Blautia* can lead to increased GVHD mortality.^42^ D'Amico et al. confirmed that compared to patients receiving PN, the gut microbiota of patients receiving EN after transplantation recovered more rapidly. In addition, the abundance of *Blautia*, *Dorea*, and *Bacteroidaceae* increased in patients receiving EN, while the abundance of *Faecalibacterium* decreased significantly in patients receiving PN. A further metabolomic analysis showed that SCFAs, including butyrate, acetate, and propionate, were significantly enriched in patients receiving EN, while patients receiving PN showed reduced SCFA levels.^125^ Finally, another study confirmed that patients receiving EN had a higher abundance of SCFA‐producing bacteria—including *Ruminococcus bromii* and several *Faecalibacterium praunitzii* strains—relative to patients receiving PN.^126^

Taken together, the studies mentioned above support the recommendation that EN rather than PN should be used to treat allo‐HSCT patients. According to experimental evidence, EN can better maintain the integrity of intestinal mucosa, regulate the composition of gut microbiota, reduce the incidence of GVHD, and improve the prognosis of GVHD patients. Patients receiving allo‐HSCT should therefore be encouraged to optimize oral intake, even during nutritional support. A randomized, prospective, multicenter study is currently evaluating the effects of different nutritional regimens on GVHD and TRM (NCT01955772). This is valuable, and additional multicenter randomized controlled studies are needed to better determine the best nutrition management method for allo‐HSCT patients.

## 15 FECAL MICROBIOTA TRANSPLANTATION

FMT involves transplantation of functional bacteria found in the feces of healthy people into the intestinal tract of recipient patients.^127^ This procedure can directly change the composition of the gut microbiota of the host; it does so by rebuilding the intestinal microbiome balance, repairing the intestinal mucosal barrier, controlling the inflammatory response, regulating the immune response, and treating intestinal and extraintestinal diseases.^128^ Since the gut microbiota plays an important role in the pathogenesis of GVHD, FMT has been studied as a potential therapeutic intervention. Currently, several studies have confirmed that FMT shows good clinical efficacy and safety in steroid‐resistant/refractory GVHD (Table 4). Moreover, the European Society for Blood and Marrow Transplantation included FMT as a treatment for steroid‐resistant/refractory GVHD patients in 2020.^129^

**TABLE 4:** Summary of recent FMT clinical trials in GVHD patients.

Patients	Donor	Administration route	Efficacy	Other response	Adverse events (AEs)
4 patients with gut GVHD (steroid‐resistant: 3; steroid‐dependent: 1)^136^	Related or spouse	Nasogastric tube	CR:3 PR:1	FMT increases peripheral effect regulatory T cells	No severe AEs
3 patients with refractory GI‐aGVHD^177^	Unrelated or Relative	Colonoscopy	CR:2 PR:1	FMT restored the diversity of intestinal flora in patients	One bacteremia (probably not related)
8 patients with steroid‐resistant GVHD^178^	Unrelated	Nasoduodenal tube	CR:5 PR:1	Compared to those who did not receive FMT, patients received FMT achieved a higher progression‐free survival (PFS)	No serious AEs
7 patients with steroid resistant/dependent GVHD (steroid‐resistant: 6; steroid‐dependent: 1)^179^	Unrelated	Oral capsules	CR:2	FMT was associated with the introduction of new bacteria and an increase in bacterial diversity in the recipient's stool	2 bacteremia (probably not related)
1 patient with steroid‐resistant grade IV gut GVHD^180^	Unrelated	Oral capsules	CR:1	The diversity of intestinal microbiota increased after FMT	No serious AEs
15 patients with steroid‐refractory or steroid‐dependent GVHD^131^	Unrelated	Nasoduodenal tube	CR:11	The fecal microbial composition of CR patients resembled that of the donor the most after FMT	No serious AEs
15 patients with steroid‐refractory or steroid‐dependent, acute or late‐onset acute intestinal GVHD (steroid‐refractory: 6; steroid‐dependent: 9)^132^	Unrelated	Nasoduodenal infusion	CR:10	Increased α‐diversity of gut microbes after FMT, the abundance of butyrate‐producing bacteria, including *Clostridium* and *Blautia* species	No serious AEs
27 patients with gastrointestinal GVHD (FMT group:19; placebo group: 8)^181^	Unrelated or relative	Gastroscopy, nasointestinal tube, or oral capsules	CR:8 PR:8	Compared to placebo group, overall bacterial mass, bacterial numbers of *Bifidobacterium* spp, *Escherichia coli* and *Bacteroides fragilisgr* were higher after FMT	No data
9 patients with severe, treatment refractory GI‐GVHD^133^	Unrelated or sibling	Endoscopically	CR:4	The microbiota characteristics in responders were more significantly similar to those of FMT donors	No serious AEs
41 patients with grade IV steroid‐refractory GI‐GVHD (FMT group: 23; Control group:18)^135^	Unrelated	Nasoduodenal/nasogastric tube	CR:13 PR:3	The event‐free survival and overall survival were higher; the mortality rate was lower in FMT group. FMT reconstructs important symbiotic groups of patients (*Spirillaceae, Ruminococcus* and *Bacteroide*s)	1 thrombocytopenia, 1 cardiac event
76 patient with steroid refractory GI‐aGVHD (HERACLES study:24; EAP study:52)^134^	Unrelated	Nasogastric tube or enema	CR:29 VGPR:14 PR:5	OS was significantly higher in responding patients compared to non‐responding. The *α* diversity index was higher in R patients than in NR patients	5 serious AEs in 2 patients
13 patients with GVHD colonized by drug‐resistant bacteria (aGVHD:11; cGVHD:3)^138^	Unrelated	Nasoduodenal tube	CR:6 PR:2	cGVHD treated with FMT achieved stabilization or improvement of organ disease. 11 patients were observed antibiotic‐resistant bacteria decolonization or partial decolonization	1 septic shock, 1 sepsis, 1 norovirus mediated gastrointestinal infection
4 patients with steroid refractory GI‐aGVHD^182^	Unrelated	Nasoduodenal tube	CR:3	/	No serious AEs
11 patients with steroid refractory GI‐aGVHD^183^	Unrelated	Oral capsules or nasojejunal tube	CR:9 PR:2	FMT can increase the diversity of intestinal microbiome. FMT led to an increase of *Ruminococcaceae, Bacteroidaceae* and *Lachnospiraceae*, an decrease of *Akkermansiaceae, Enterococcaceae* and *Veillonellaceae*	No serious AEs
21 patients with intestinal steroid‐refractory GI‐GVHD^139^	Unrelated	Oral capsules	CR:10 PR:5	The levels of inflammatory cytokines as well as T cells and NK cells activation declined. The diversity of the intestinal microbiota was improved in responders	Viral reactivations and severe cytopenia

There is increasing evidence that FMT has good efficacy and safety when treating steroid‐dependent/resistant aGVHD patients. Kakihana et al. first applied FMT to treat steroid‐resistant/dependent intestinal aGVHD in 2016 and found that all patients (i.e., four cases) responded to FMT treatment, with three complete responses (CR), and one partial response (PR). Patients with steroid‐resistance all showed improvement in GI symptoms within a few days, and no serious adverse effects were observed during treatment. Moreover, this study also confirmed that FMT can increase the number of peripheral effector regulatory T cells (eTreg), which have been reported to be prognostic cellular biomarkers for aGVHD. Gut microbial composition analysis showed FMT can increase the diversity of gut microbiota in aGVHD patients, among which the abundance of beneficial bacteria including *Bacteroides*, *Lactobacillus*, *Bifidobacterium*, and *Faecalibacterium* was increased in response to FMT.^130^ Several subsequent studies have further evaluated the efficacy and safety of FMT; they have shown that 50%–85% of patients can achieve CR, and that FMT can prolong the median OS and event‐free survival (EFS) of patients, reduce GVHD‐related mortality, regulate intestinal immunity (i.e., reduce the infiltration of Th17 and CD8^+^ T cells, and increase the number of Treg and type 3 innate lymphocytes (ILC3)), and change the diversity and composition of the gut microbiota.^131^, ^132^, ^133^, ^134^, ^135^ Therefore, FMT appears to be safe and effective, and is expected to be a potential treatment option for aGVHD.^136^

Due to the long‐term use of broad‐spectrum antibiotics, as well as the destruction of the gut microbiota caused by pretreatment regimens, allo‐HSCT patients are often colonized by multidrug‐resistant bacteria (MDRB), which presents a major treatment challenge. Battipaglia et al. confirmed that FMT is safe and effective for treating allo‐HSCT patients carrying MDRB; in that study, seven of ten patients were successfully decolonized after FMT. Although one patient experienced constipation and two patients experienced grade I diarrhea after FMT, but no other adverse events were observed.^137^ Bilinski et al. conducted a prospective multicenter study to assess the effectiveness and safety of FMT in patients with GVHD who were colonized by antibiotic‐resistant bacteria (ARB). According to the results, 11 of 14 patients had ARB decolonized or partially decolonized after FMT. Among aGVHD patients, the ORR was 57% (8/14 FMTs), including 42% (6/14) reaching CR. Moreover, the median OS of responders and non‐responders was 332 and 66 days, respectively (HR = 0.18, 95 %CI: 0.03–0.93, *p* < 0.005). After FMT treatment, both patients with cGVHD showed stabilization or improvement of their organ disease and were still alive at the final follow‐up.^138^ Therefore, FMT appears to be highly effective for the treatment of GVHD and for decolonization of the GI tract from ARB.

The combination of FMT and other protocols has also demonstrated favorable efficacy and safety outcomes in patients with steroid‐refractory aGVHD. For example, a recent study by Liu et al. evaluated the effectiveness of FMT in combination with ruxolitinib as a salvage therapy for intestinal steroid‐refractory aGVHD after allo‐HSCT. According to their results, the ORR on Day 28 of the combined regimen was 71.4%, with 10 patients reaching CR and five patients reaching PR. The durable overall response in responders on Day 56 was 80%, the estimated 6‐month OS was 57.1%, and the EFS was 52.4%. In addition, the authors observed declines in the levels of inflammatory cytokines (mainly including IL‐2, IL‐17A, IL‐4, IL‐6, and IL‐10), T cells, and in NK cell activation. In addition, the diversity of gut microbiota was higher in patients who responded to combination therapy, while the proportion of *Lactobacillus* was increased in CR patients and the abundance of *Escherichia* was lower following treatment. The most frequent adverse events recorded were viral reactivation (61.9%) and severe cytopenia (grades 3–4, 81.0%). Therefore, this study confirmed that FMT combined with ruxolitinib is an effective treatment for intestinal SR‐aGVHD following HSCT.^139^

However, the safety of FMT may need further to be fully validated. FMT involves the transfer of feces from the donor to the recipient, so there is a risk of infectious disease. Two previous independent clinical trials in which the donors had not been screened for multidrug‐resistant organisms reported the spread of extended‐spectrum beta‐lactamase (ESBL) with adverse consequences. Specifically, both patients displayed ESBL‐producing *Escherichia coli* bacteremia after undergoing FMT, and one of the patients died.^140^ Furthermore, a worldwide analysis of FMT‐related adverse events from 129 studies performed between 2000 and 2020 showed that FMT‐related serious adverse events (SAEs), including infection and deaths, occurred in 1.4% of patients who underwent FMT. Moreover, all reported FMT‐related SAEs occurred in patients with mucosal barrier injury. Therefore, researchers and clinicians should pay attention to the potential risks of FMT for treating GI‐aGVHD.^141^ Another study confirmed a high incidence of bloodstream infections (BSIs) following FMT for GVHD (i.e., in 22 events out of 33 patients).^142^ Therefore, it is still necessary to pay full attention to the safety of FMT in clinical practice, and donor screening and careful benefit risk assessments are required when designing FMT research protocols.^143^

In conclusion, although some of the above studies confirm that FMT shows good efficacy and safety in patients with GVHD, where it increases the diversity of gut microbiota and changes the composition of gut microbiota. However, most of these studies are single‐center, small‐sample studies, and focused mainly on applications to patients with aGVHD, while studies of patients with cGVHD were limited. In addition, the specific mechanisms by which FMT affects GVHD patients remain unclear. Therefore, more prospective, multicenter studies are required to evaluate the safety and efficacy of FMT for patients with GVHD (Table 5).

**TABLE 5:** Ongoing clinical trials for the treatment and prevention of GVHD.

Trial title	Disease state(s)	Intervention	Phase	Clinicaltrials.gov identifier
*Nutritional intervention*				
Study on intelligent nutrition support therapy for hematopoietic stem cell transplantation recipients	Allo‐HSCT	Parenteral nutrition Enteral nutrition	NA	NCT05590091
Randomized prospective multicenter study to compare enteral nutrition with parenteral nutrition as feeding support in patients presenting with malignant hemopathy who underwent an allogeneic HSC transplantation (NEPHA)	Allo‐HSCT	Enteral nutrition alanyl‐glutamin, Dipeptiven; Parenteral nutrition	3	NCT01955772
*FMT*				
Fecal microbiota transplantation for treatment of refractory graft‐versus‐host disease‐a pilot study	Steroid‐resistant GI‐related aGVHD	FMT	Pilot	NCT03549676
Prospective study of FMT for acute intestinal GVHD After allo‐HSCT	Steroid‐resistant/dependent intestinal aGVHD	FMT	NA	NCT04711967
FMT in high‐risk acute GVHD after ALLO HCT	High‐risk aGVHD	FMT	1	NCT04139577
Fecal microbiota transplant (FMT) capsule for improving the efficacy of GI‐aGVHD	Glucocorticoid‐refractory aGVHD	FMT	1	NCT05094765
Fecal microbiota transplantation for the treatment of severe acute gut graft‐versus‐host disease	High‐risk or steroid refractory aGVHD	FMT	1	NCT04280471
FMT for Steroid resistant gut acute GVHD	aGVHD	FMT	1	NCT04285424
Fecal microbiota transplantation for steroid resistant and steroid‐dependent gut acute graft‐versus‐host disease	Steroid‐resistant or steroid‐dependent gut aGVHD	FMT	1	NCT03214289
Efficacy and safety of FMT capsule treating steroid‐refractory GI‐aGvHD	Steroid refractory GI‐aGVHD	FMT	NA	NCT04622475
Fecal microbiota transplant and dietary fiber supplementation for the treatment of gut graft‐versus‐host disease	Intestinal aGVHD	FMT, dietary fiber supplementation	1	NCT05067595
Fecal microbiota transplantation in gut aGVHD treated	Gut aGVHD	FMT	None	NCT03148743
Efficacy and safety of auto‐FMT in preventing aGVHD	aGVHD	Autologous fecal bacteria	NA	NCT04745221
Fecal microbiota transplantation for steroid resistant/dependent acute GI GVHD	Steroid resistant/dependent acute intestinal GVHD	FMT	2	NCT03812705
Fecal microbiota transplantation in aGVHD after ASCT	Steroid refractory GI‐aGVHD	FMT	3	NCT03819803
MaaT013 as Salvage therapy in ruxolitinib refractory GI‐aGVHD patients	Steroid refractory GVHD	MaaT013	3	NCT04769895
Fecal microbiota transplantation with ruxolitinib and steroids as an upfront treatment of severe acute intestinal GVHD (JAK‐FMT)	Gastrointestinal aGVHD	FMT+ruxolitinib+steroids	1/2	NCT04269850
*Probiotics*				
Lactobacillus plantarum in preventing acute graft‐versus‐host disease in children undergoing donor stem cell transplant	HSCT recipient	Lactobacillus plantarum strain 299 or 299v	3	NCT03057054
CBM588 in improving clinical outcomes in patients who have undergone donor hematopoietic stem cell transplant	HSCT recipient	Clostridium butyricum CBM 588 probiotic strain	1	NCT03922035
*Prebiotics*				
Prebiotic galacto‐oligosaccharide and acute GVHD	aGVHD	Galacto‐oligosaccharide	1/2	NCT04373057
Gluten‐free diet in preventing graft‐versus‐host disease in patients undergoing donor stem cell transplant	HSCT recipient	Gluten‐free diet (GFD)	NA	NCT03102060
Dietary manipulation of the microbiome‐metabolomic axis for mitigating GVHD in allo HCT patients	HSCT recipient	Potato starch	2	NCT02763033
Effects of prebiotics on gut microbiome in patients undergoing HSCT	HSCT recipient	Prebiotic foods/drinks	NA	NCT04629430
The use of a prebiotic to promote a healthy gut microbiome in pediatric stem cell transplant recipients	HSCT recipient	Prebiotics: inulin	NA	NCT04111471
Oral supplementation of 2′‐fucosyllactose in allogeneic bone marrow transplant recipients	HSCT recipient	2′‐fucosyllactose	1/2	NCT04263597
High‐dose vitamin A in preventing gastrointestinal GVHD in participants undergoing donor stem cell transplant	HSCT recipient	Vitamin A compound	NA	NCT03719092
Fructooligosaccharides in treating patients with blood cancer undergoing donor stem cell transplant	HSCT recipient	Fructooligosaccharides orally	1	NCT02805075
*Antibiotics*				
Optimization of antibiotic treatment in hematopoietic stem cell receptors (optimbioma)	HSCT recipient	Optimization/antibiotic strategy	NA	NCT03727113
Rifaximin for infection prophylaxis in hematopoietic stem cell transplantation	HSCT recipient	Rifaximin	1	NCT03529825
Gut decontamination in pediatric allogeneic hematopoietic	HSCT recipient	Vancomycin‐polymyxin B	2	NCT02641236
Choosing the best antibiotic to protect friendly gut bacteria during the course of stem cell transplant	HSCT recipient	Piperacillin‐tazobactam; cefepime	2	NCT03078010
Antibacterial prophylaxis versus no prophylaxis for hematological malignancies patients before allo‐HSCT	HSCT recipient	Imipenem	2/3	NCT03733340

## 16 PROBIOTICS

Probiotics refer to living microorganisms that can bring health benefits to a host when internalized. Probiotics can refer to a single strain or a mixture of strains, and can improve the composition of gut microbiota, control the regulation the immune function of the human body, and prevent or alleviate the occurrence of a variety of diseases.^144^, ^145^ Current research on probiotic interventions often focuses on the biological use of *Bifidobacterium* and *Lactobacillus*.^146^

Probiotic‐based therapies have been shown to be able to regulate the composition of gut microbiota and improve the prognosis of GVHD. Gerbitz et al. used a mouse model to demonstrate that oral administration of the probiotic *Lactobacillus rhamnosus* GG before and after transplantation can reduce intestinal inflammation and bacterial translocation, thereby reducing the severity of aGVHD and improving survival.^147^ However, in a randomized controlled trial, supplementation with *Lactobacillus rhamnosus* GG in allo‐HSCT patients did not appear to significantly alter the gut microbiome or provide protection against GVHD.^148^ This may be due to the fact that humans have human‐specific intestinal mucosal colonization against transient probiotic colonization, which may obscure some potential benefits of probiotic treatments.^149^ Jenq et al. demonstrated that an ampicillin treatment targeting *Lactobacillales* can lead to aGVHD exacerbation in a mouse model, and that reintroduction of *Lactobacillales* following ampicillin treatment can reduce aGVHD mortality and severity. This may occur because *Lactobacillales* can reduce the severity of aGVHD by preventing the expansion of *Enterococcus*.^33^ In another study, Ladas et al. confirmed that the application of another probiotic, which contained *Lactobacillus plantarum* (LBP), is safe and reasonably tolerated in children and adolescents undergoing allo‐HSCT.^150^

Given this evidence, probiotic therapy may be an effective treatment to improve the severity and mortality of GVHD, but requires further validation in subsequent prospective studies. Studies are currently evaluating the efficacy of probiotics such as *Lactobacillus plantarum* (NCT03057054) and *Clostridium butyricum MIYAIRI 588* (CBM588) (NCT03922035) in GVHD patients (Table 5).

## 17 PREBIOTICS

Prebiotics are dietary supplements that can help improve the growth and development of beneficial bacteria, inhibit harmful bacteria, and increase the production of beneficial microbiota‐derived metabolites, including SCFAs, via bacterial fermentation.^3^, ^151^ Prebiotics have been shown to improve the prognosis of GVHD by maintaining intestinal integrity and modulating immune response. Lyama et al. used a retrospective study to demonstrate that nutritional supplements consisting of glutamine, fiber, and oligosaccharides reduced the severity of mucosal injury following allo‐HSCT.^152^ Galactooligosaccharides (GOS) are a widely studied prebiotic ingredient that are used as a dietary supplement to reduce inflammatory GI symptoms, promote intestinal barrier function, increase NK cell activity, and regulate cytokine activity.^153^, ^154^ In a recent study using GVHD model mice, Holmes et al. confirmed that exogenous supplementation of GOS can improve the survival rate of allo‐HSCT mice and reduce the severity of GVHD. Interestingly, GOS only improved the allo‐HSCT prognoses of antibiotic‐treated mice, which suggests that prebiotics could counter the harmful effects of antibiotics on commensal gut microbiota and clinical outcomes. In addition, this study also confirmed that the supplementation of GOS could improve the composition of gut microbiota, since it showed that the probiotic treatment was associated with reduced abundance of *Bacteroidaceae* and *Bacteroidales_S24‐7_group* as well as increased abundance of *Porphyromonadaceae* was increased.^154^, ^155^ Furthermore, *Porphyromonadacea* is involved in butyrate production, and has been found to reduce the severity of GVHD.^156^, ^157^ Therefore, this study suggests that prebiotic therapy may be an adjunct therapy for the prevention of GVHD. Studies are currently evaluating the efficacy of prebiotic regimens including potato starch (NCT02763033), a gluten‐free diet (NCT03102060), fructose oligosaccharides (NCT02805075), GOS (NCT04373057), inulin (NCT04111471), and 2′‐fucosyllactose (NCT04263597) (Table 5) in GVHD patients.

## 18 ANTIBIOTICS

Preconditioning regimens can lead to neutropenia, which result in oral and gastrointestinal mucositis that increases the risk of bacterial translocation, in turn leading to increased risk of various infections.^158^ Therefore, allo‐HSCT patients are often given prophylactic or empirical antibiotics to prevent and treat bacterial infections.^159^ Early studies using mouse models have shown that sterile environment feeding^35^ or antibiotic‐mediated intestinal purification^36^ can alleviate aGVHD symptoms. However, subsequent studies have not confirmed the benefits of intestinal purification in reducing GVHD.^160^ In addition, many studies have shown that the use of antibiotics affects the composition and diversity of gut microbiota, which leads to different clinical results and can either increase or decrease the risk of GVHD.^3^, ^160^, ^161^, ^162^ Therefore, the rational deployment of antibiotic treatments is needed in clinical practice.

Some broad‐spectrum antibiotics such as piperacillin‐tazobactam, meropenem, and imipenem‐cilastatin can reduce the abundance of *Clostridiales, Bifidobacteriales, Blautia*, and *Lactobacillus*, but increase the abundance of *Akkermansia muciniphila*, *Enterococcus*, and *Bacteroides thetaiotaomicron*.^42^, ^163^, ^164^, ^165^ Another study showed that exposure to broad‐spectrum antibiotics was significantly associated with increased incidence of GVHD‐ and GVHD‐related mortality.^165^, ^166^, ^167^, ^168^, ^169^ A retrospective study confirmed that imipenem‐cilastatin and piperacillin‐tazobactam antibiotic treatments for neutropenic fever were associated with increased GVHD‐related mortality after 5 years. In addition, mice treated with imipenem‐cilastatin showed increased abundance of *Akkermansia muciniphila* and reduced colonization of *Clostridiales* in the gut.^163^ Furthermore, Tanaka et al. showed that the abundance of *Bifidobacteriales* and *Clostridiales* decreased in patients that received piperacillin‐tazobactam or carbapenems between 7 days before to 28 days after HSCT. In contrast, patients who received piperacillin‐tazobactam or carbapenems experienced a higher risk of acute gut/liver GVHD and GVHD‐related mortality.^164^

Specific narrow‐spectrum antibiotics such as cefepime, aztreonam, and rifaximin have been associated with reduced GVHD severity. For example, Shono et al. confirmed that treatment with cefepime and aztreonam was significantly associated with a reduced risk of GVHD‐related mortality.^163^ Rifaximin is a rifamycin derivative, and can retain the structure of the colonic microbiota and exert anti‐inflammatory activity.^170^ In a retrospective study, Weber et al. confirmed that the use of rifaximin reduced the negative effects of systemic antibiotics on microbial composition relative to patients taking a ciprofloxacin/metronidazole treatment. Moreover, patients treated with rifaximin often showed higher urinary 3‐IS levels and a lower abundance of *Enterococcus* and rifaximin can reduce the incidence of intestinal GVHD, reduce GVHD‐related mortality, and improve OS.^171^ Another study demonstrated that patients treated with rifaximin alone or rifaximin combined with systemic antibiotics showed higher 3‐IS levels, a higher abundance of *Clostridium cluster XIVa* (CCXIVa), a higher Shannon index, a lower incidence of severe GI GVHD, a lower TRM, and longer OS.^162^ Given these results, the evidence suggests that rifaximin permits a higher gut microbiome diversity, even in the presence of systemic broad‐spectrum antibiotics.

In addition, the duration of antibiotic use is also associated with differences in aGVHD response. A retrospective study evaluated the effect of antibiotic use timing on gut microbiota composition and prognosis, and their results showed that early exposure to antibiotics (i.e., between Day −7 and Day 0 relative to the allo‐HSCT procedure) significantly reduced the abundance of symbiotic bacteria *Clostridiales* and increased GVHD‐related mortality.^172^

Taken together, these studies confirm that different antibiotic regimens and timing of use treatments following allo‐HSCT are associated with different clinical outcomes. Specifically, the use of broad‐spectrum antibiotics is associated with increased incidence and severity of GVHD in multiple centers, while some narrow‐spectrum antibiotics, such as rifaximin, are associated with decreased incidence and severity of GVHD. Therefore, rational selection of antibiotic treatments after allo‐HSCT may be able to reduce the incidence and severity of GVHD. Further multicenter, prospective studies are required to optimize the use of antibiotics in allo‐HSCT patients to both prevent bacteremia or sepsis while limiting intestinal microbiome damage, thereby reducing the incidence and mortality of GVHD.

## 19 CONCLUSION

In conclusion, there is increasing empirical evidence that gut microbiota and microbiota‐derived metabolites play important roles in the occurrence and development of GVHD. Moreover, their dysregulation appears to be significantly associated with poor prognosis. A series of therapeutic strategies to regulate the gut microbiota via nutritional support, FMT, probiotics, prebiotics, and adjustment of antibiotic use may assist in the clinical prevention and treatment of GVHD. However, the specific mechanisms involved in the regulation of gut microbiota and microbiota‐derived metabolites in GVHD remain unclear. Therefore, it is still necessary to further explore the role of gut microbiota and microbiota‐derived metabolites in GVHD in future research. This can elucidate the real effects of gut microbiota on GVHD, and facilitate the identification of a safe and effective treatment strategy for regulating the damaged microbiota of GVHD patients, thereby improving their prognosis.

## AUTHOR CONTRIBUTIONS

**XiaoYan Yue:** Conceptualization (equal); investigation (equal); software (equal); visualization (equal); writing – original draft (lead). **Hongyu Zhou:** Conceptualization (equal); writing – original draft (equal). **ShuFen Wang:** Writing – review and editing (equal). **Xu Chen:** Investigation (equal); writing – original draft (equal). **HaoWen Xiao:** Funding acquisition (lead); project administration (lead); supervision (equal); validation (equal); writing – review and editing (lead).

## FUNDING INFORMATION

This study was supported by the National Natural Science Foundation of China (81870136, 82170141) and the Natural Science Foundation of Zhejiang Province (LXZ22H080001).

## CONFLICT OF INTEREST STATEMENT

The authors declare no competing financial interests.

## CONSENT FOR PUBLICATION

All authors agree with the final version of the manuscript and give their consent for its publication.

## Data Availability

Not applicable.
